# Editorial: Invasive fungal disease in the immunocompromised host/Research Topic proceedings of the mycology 2021 meeting

**DOI:** 10.3389/fcimb.2022.1033739

**Published:** 2022-09-29

**Authors:** Samir Agrawal, Neil Gow, Duncan Wilson, Lewis White

**Affiliations:** ^1^ Haemato-Oncology, Barts Health National Health Service (NHS) Trust, London, United Kingdom; ^2^ Immunobiology, Blizard Institute, Queen Mary University of London, London, United Kingdom; ^3^ Medical Research Council Centre for Medical Mycology, University of Exeter, Exeter, United Kingdom; ^4^ Medical Research Council (MRC) Centre for Medical Mycology, College of Life and Environmental Sciences, University of Exeter, Exeter, United Kingdom; ^5^ Public Health Wales (PHW) Mycology Reference Laboratory, University Hospital of Wales, Cardiff, United Kingdom

**Keywords:** invasive fungal disease, snps, fungal diagnostics, cystic fibrosis, ABPA, bronchial asthma, central bronchiectasis

This Frontiers Research Topic presents updates of work shown at Mycology 2021 - an entirely virtual meeting organised by the Fungal Update symposium team and the British Society for Medical Mycology –with 206 attendees in 20 countries and 39 abstracts accepted. The programme can be accessed on-line at: https://portalapp.bsac.eventsair.com/VirtualAttendeePortal/mycology-2021/event-page?Token=RXWb4XaCN8FX9WNH0B7EnL48ywJanzRw4ujZpUe4OQjTMVoFUAjjxRZJmbWrysdL. The conference brought together a world community of clinical and basis research scientists to discuss and debate contemporary topics in the diagnosis, treatment of fungal infections and the latest advances in discovery based research in the cell, molecular and immunology of fungal pathogens.

Please do have a look – it is a great virtual experience!

On day 1 of Mycology 2021, IFD management at three European haematology centres was presented during a parallel session. Here, Busca et al update their “Turin experience” on IFD management in the allogeneic hematopoietic stem cell transplantation setting. While mould-active prophylaxis is standard management in many centres, Busca et al. found in a large cohort of patients (n=593) that non-mould active prophylaxis combined with a diagnostic strategy is feasible. The findings are in keeping with a similar approach in the setting of remission induction therapy for acute myeloid leukaemia (De Kort et al., 2022). There are considerable potential benefits: reduced exposure to antifungal drugs; reduction in the associated drug toxicities; decreased development of drug resistance; reduced drug-drug interactions between antifungals and a wide range of essential transplant-related drugs.

The diagnosis of IFD remains a key clinical challenge and this was addressed extensively in the “Diagnostics” session on Day 1(14:40) of Mycology 2021. White and Price review here a novel approach to combining diagnostics with host genetic predisposition (using single nucleotide polymorphisms) and speculate as to how algorithms combining risk prediction and biomarker test results could aid the diagnosis of invasive aspergillosis.

Two sessions at Mycology 2021 (Day 1 16:00 and Day 2 11:20) addressed fungal infection in patients with cystic fibrosis (CF). In this Research Topic, van Rhijn et al add another dimension to the complexity of factors affecting the risk of pulmonary infections in patients with CF. The authors identify the impact of weather conditions on fungal spore counts. Simple preventative measures appeared to be associated with decreased spore levels in the hospital environment and hence potentially reduced risk of a range of diseases associated with *Aspergillus fumigatus* in patients with CF. Another population of patients at risk of allergic bronchopulmonary aspergillosis (ABPA) are those with asthma. Agarwal et al report on simplified criteria that may aid diagnosis of ABPA and hence its management.

Throughout the two days of Mycology 2021 both plenary and parallel sessions were dedicated to basic research in mycology. In this Research Topic, Rahman et al produce further data from Rahman et al elucidating the key transcription factors involved in *A. fumigatus*-induced damage to lung epithelium. The opportunity for researchers and clinicians to rub shoulders, exchange ideas, learn from each other and collaborate on future projects has always been a key feature of the Fungal Update meetings. In fact, Fungal Update 2022 was the first in-person meeting since the COVID-19 pandemic and took place in June of this year. This meeting can also be accessed on-line at https://mycologyconference.co.uk/. We look forward to continuing this successful approach in future meetings.

## Author contributions

All authors listed have made a substantial, direct, and intellectual contribution to the work and approved it for publication.

## Conflict of interest

The authors declare that the research was conducted in the absence of any commercial or financial relationships that could be construed as a potential conflict of interest.

## Publisher’s note

All claims expressed in this article are solely those of the authors and do not necessarily represent those of their affiliated organizations, or those of the publisher, the editors and the reviewers. Any product that may be evaluated in this article, or claim that may be made by its manufacturer, is not guaranteed or endorsed by the publisher.
